# Effects of Natural Products on Enzymes Involved in Ferroptosis: Regulation and Implications

**DOI:** 10.3390/molecules28237929

**Published:** 2023-12-04

**Authors:** Hua-Li Zuo, Hsi-Yuan Huang, Yang-Chi-Dung Lin, Kun-Meng Liu, Ting-Syuan Lin, Yi-Bing Wang, Hsien-Da Huang

**Affiliations:** 1 School of Medicine, The Chinese University of Hong Kong, Shenzhen, Shenzhen 518172, China; huanghsiyuan@cuhk.edu.cn (H.-Y.H.); yangchidung@cuhk.edu.cn (Y.-C.-D.L.); 222050501@link.cuhk.edu.cn (T.-S.L.); 222051029@link.cuhk.edu.cn (Y.-B.W.); 2 Warshel Institute for Computational Biology, The Chinese University of Hong Kong, Shenzhen, Shenzhen 518172, China; 3 Center for Medical Artificial Intelligence, Qingdao Academy of Chinese Medical Sciences, Shandong University of Traditional Chinese Medicine, Qingdao 266112, China; liukunmeng@sdutcm.edu.cn

**Keywords:** ferroptosis, natural product, enzyme, glutathione metabolism, oxidative stress, lipid metabolism

## Abstract

Ferroptosis is a form of regulated cell death that is characterized by the accumulation of iron-dependent lipid peroxides. The regulation of ferroptosis involves both non-enzymatic reactions and enzymatic mechanisms. Natural products have demonstrated potential effects on various enzymes, including GPX4, HO-1, NQO1, NOX4, GCLC, and GCLM, which are mainly involved in glutathione metabolic pathway or oxidative stress regulation, and ACSL3 and ACSL4, which mainly participate in lipid metabolism, thereby influencing the regulation of ferroptosis. In this review, we have provided a comprehensive overview of the existing literature pertaining to the effects of natural products on enzymes involved in ferroptosis and discussed their potential implications for the prevention and treatment of ferroptosis-related diseases. We also highlight the potential challenge that the majority of research has concentrated on investigating the impact of natural products on the expression of enzymes involving ferroptosis while limited attention is given to the regulation of enzyme activity. This observation underscores the considerable potential and scope for exploring the influence of natural products on enzyme activity.

## 1. Introduction

Several instances of ferroptosis were documented prior to 2012, when a comprehensive molecular comprehension of ferroptosis was established. Ferroptosis, a type of regulated cell death (RCD), is governed by both non-enzymatic (Fenton reaction) and enzymatic mechanisms [1]. It is morphologically, genetically, and biochemically distinct from other forms of RCD, such as apoptosis, necroptosis, and autophagy. This process relies on iron and is characterized by a reduction in glutathione levels and the accumulation of lipid peroxides, which are lipids that have undergone oxidative degradation [2,3]. The intricate process of ferroptosis is governed by a multitude of biological mechanisms, encompassing numerous enzymes and pertinent molecules such as iron, amino acids, and polyunsaturated fatty acids (PUFAs). Additionally, the interplay between glutathione (GSH), NADPH, coenzyme Q10 (CoQ10), and phospholipids further contributes to the regulation of ferroptosis, impacting various cellular metabolic pathways including redox homeostasis, iron homeostasis, and lipid metabolism [4].

Ferroptosis plays a pivotal role in tumor suppression and can reverse drug resistance in cancer therapy [5,6], and it has also been implicated in various non-cancer pathological conditions, such as neurodegeneration [7], ischemia-reperfusion injury [8], and COVID-19 [9].

Having grown rapidly in recent years, ferroptosis has gradually established an interaction with natural products, which are conventional resources for drug discovery and modification. Enzymatic reactions are interconnected, and the delicate equilibrium between them ultimately determines whether a cell undergoes ferroptosis or manages to survive. Previous studies have demonstrated the therapeutic potential of natural products in the treatment and prevention of various diseases, with their effectiveness attributed to their ability to induce apoptosis, inhibit angiogenesis, suppress cell growth, regulate inflammation, and modulate oxidative stress [10,11,12]. In addition, recent research has also begun to establish a connection between ferroptosis and natural products [13]. Consequently, it is crucial to comprehend the molecular mechanisms underlying ferroptosis and identify potential modulators derived from natural products to advance the development of novel therapeutic strategies. Botanical drugs contain a rich source of naturally bioactive compounds considered conventional sources for drug discovery and modification and can affect various cellular processes, including ferroptosis [14].

This review aims to provide a comprehensive overview of the existing literature regarding the impact of natural products on the enzymatic activities associated with ferroptosis. Specifically, we focus on the enzymes GPX4, HO-1, NQO1, NOX4, GCLC, and GCLM, which primarily contribute to glutathione metabolism and regulation of oxidative stress; and ACSL3, and ACSL4, which mainly participate in lipid metabolism. We also highlight the potential applications and challenges, current advances of the emerging regulatory mechanisms and disease relevance of ferroptosis.

## 2. Results and Discussion

### 2.1. Search Results and Study Inclusion

**Figure 1:**
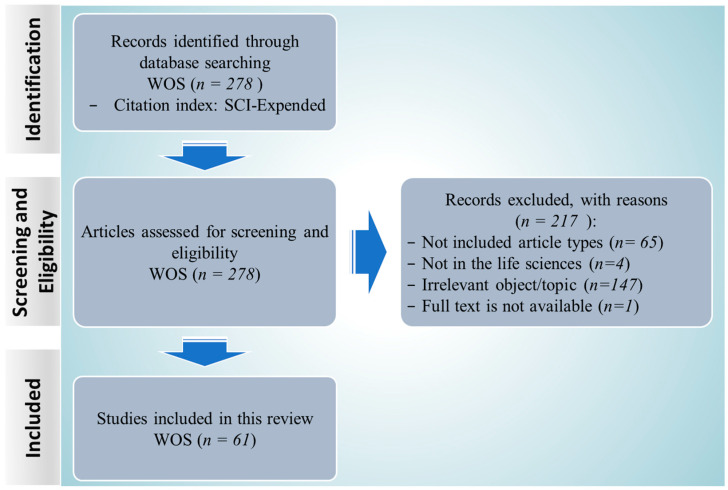
PRISMA flow diagram detailing the number of papers included at each stage and the reasons for removal.

The search results were combined and duplicates removed to include 278 papers reporting the natural products’ activity on enzymes involved in ferroptosis. After removing the sixty-five papers that were not research articles, and four papers were not concluded in the life sciences, we read the abstracts of the remaining two-hundred-and-nine articles for relevance, specifically focusing on the enzymes associated with ferroptosis as indicated in Table 1, based on prior knowledge. Subsequently, a more thorough analysis led to the exclusion of 147 articles due to their lack of relevance, and one article was excluded due to unavailability of the full text. As a result of this literature screening process, a total of 61 full-text studies were deemed suitable for inclusion in this review (Figure 1).

### 2.2. Study Characteristics

**Figure 2:**
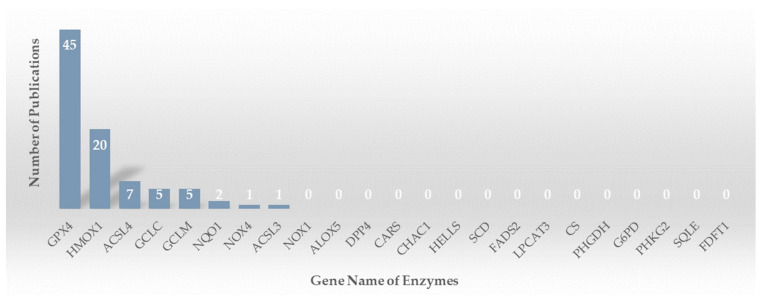
Publications reporting the activity of natural products on enzymes involving ferroptosis.

The included 61 studies mainly reported the activity of natural products on the enzymes included GPX4, HO-1 (encoded by HMOX1), ACSL4, GCLC, GCLM, NQO1, NOX4, and ACSL3 (Figure 2). GPX4 attracts considerable scholarly interest, possibly due to the fact that the concept of ferroptosis is predominantly defined as a form of cell demise dependent on iron and triggered by oxidative stress-induced dysfunction of phospholipids. GPX4, being a phospholipid hydroperoxidase, plays a crucial role in preventing lipid peroxidation and irreversible cellular demise when inactivated [38].

### 2.3. The Molecular Mechanisms of Ferroptosis

The molecular regulatory mechanisms governing ferroptosis are intricate, entailing various biological processes, including extrinsic: iron metabolism, transport of component of GSH; and intrinsic: lipid metabolism/peroxidation iron metabolism [39]. The initiation of the extrinsic pathway is facilitated by the regulation of transporters, specifically those responsible for iron and lipid transport. These transporters govern the cellular absorption of iron and the synthesis of PUFAs. The accumulation of iron and PUFAs subsequently triggers lipid peroxidation, which plays a pivotal role in the occurrence of ferroptosis. In contrast, the intrinsic pathway primarily arises from the inhibition of intracellular antioxidant enzymes, such as GPX4, which play a crucial role in safeguarding cells against oxidative harm by converting lipid hydroperoxides into their respective alcohols.

### 2.4. Enzymes-Mediated Phospholipid Peroxidation

PUFAs are prone to oxidative attack at the carbon-carbon double bonds of lipids, a process known as “lipid peroxidation”, and play a crucial role in the initiation and execution of ferroptosis [40,41]. The biosynthesis and modification of PUFAs involve the actions of two enzymes, namely ACSL4 and LPCAT3, which facilitate the incorporation of PUFA into membrane phospholipids (PL-PUFA) and the formation of phosphatidylethanolamine (PEs)-containing PL-PUFA [29]. In detail, ACSL4 catalyzes the conversion of free PUFAs, such as arachidonic acid (AA) and adrenic acid (AdA), into their corresponding CoA esters. Subsequently, LPCAT3 incorporates these PUFA-CoAs into phospholipids, leading to the production of PEs enriched with PUFA. These PEs are particularly vulnerable to peroxidation [6]. In the presence of oxidative or energetic stress, these enzymes facilitate the synthesis of PUFA-containing phospholipids and trigger ferroptosis as a means to sustain redox homeostasis.

Although the precise mechanisms by which PL-PUFAs (PE) initiate lipid peroxidation remain incompletely understood, the influence on the formation of phospholipid hydroperoxide derivatives of polyunsaturated fatty acids (PUFA-OOHs) is widely recognized to arise from both non-enzymatic and enzymatic processes [31]. These derivatives have been observed to disrupt the structural integrity of cellular membranes, disturb their characteristics, induce oxidative harm to cellular structures, and ultimately lead to ferroptotic cell death.

Furthermore, the generation of PUFA-OOHs can be facilitated by the production of reactive oxygen species (ROS) through the Fenton reaction, wherein iron acts as a catalyst to generate alkoxyl and peroxyl radicals, thereby promoting the synthesis of PUFA-OOHs (elaborated upon subsequently). Consequently, intracellular and intercellular signaling processes, along with diverse metabolic stresses, can modulate ferroptosis by regulating cellular oxidative stress and levels of ROS. For instance, the activation of antioxidant defense systems, such as peroxiredoxins, superoxide dismutase, catalase, GPXs, and Nrf2 (HO-1 regulated by Nrf2), or the production of non-enzymatic antioxidants (e.g., coenzyme Q10, tetrahydrobiopterin), can effectively reduce ROS and inhibit the occurrence of ferroptosis. Additionally, PL-PUFAs can undergo direct oxidation by lipoxygenases (LOXs), which are non-heme iron-dependent dioxygenases, and by prostaglandin-endoperoxide synthase (PTGS/COX), resulting in the generation of PUFA-OOHs and the initiation of ferroptosis.

### 2.5. The Effects of Natural Products on the Enzymes Involving Ferroptosis

A wide range of phytochemicals exhibiting various biological activities have been employed as dietary interventions for the purpose of managing human diseases. Specifically, certain bioactive components derived from different plant sources have been recognized as influential regulators of ferroptosis, both in vitro and in vivo. The natural products’ functions and their molecular mechanisms regulating enzymes involving ferroptosis were summarized, as shown in Table 2.

### 2.6. The Effects of Natural Products on the Enzymes Involving Glutathione Metabolic Pathway

#### 2.6.1. GPX4

GPX4, a phospholipid hydroperoxidase, is crucial in safeguarding cells from membrane lipid peroxidation. It is classified within the glutathione peroxidase family, encompassing eight recognized mammalian isoenzymes (GPX1-8). The primary function of GPX4 involves the reduction of hydrogen peroxide, organic hydroperoxides, and lipid peroxides, utilizing reduced glutathione as a substrate. This enzymatic activity is essential for cellular defense against oxidative stress [40]. The equilibrium between reduced glutathione (GSH) and oxidized glutathione (GSSG) is a crucial intracellular antioxidant system that plays a critical role in cell survival. Disruption of this balance leads to the accumulation of ROS and ultimately results in cell death. It is worth noting GPX4 utilizes GSH as a cofactor to convert potentially harmful PUFA-OOHs into non-toxic lipid alcohols (PUFA-OHs). Consequently, the inhibition of GPX4 through direct pharmacological inhibitors, or the depletion of GSH induces excessive lipid peroxidation and triggers ferroptotic cell death.

In previously reported studies, the most focused diseases, included various cancers (breast cancer, colorectal cancer, gastric cancer, liver cancer, lung cancer, esophageal cancer), myocardial infarction/ischemic stroke, Alzheimer’s disease (AD), or insulin secretion dysfunction, etc. Additionally, most of the natural products’ modulation on GPX4 was evaluated by mRNA or protein expression level. For disease treatment, the mRNA or protein levels of GPX4 are expected to be lower or down-regulated, when designed natural products’ treatment is given, consistent with most of the relevant research, as detailed information listed in Table 2.

What is more, there were some, though only a small part of these studies, that also investigated the on-target enzymatic activity or direct binding potential. The cellular glutathione peroxidase assay kit was used to evaluate the enzymatic activity of GPX4 to investigate the effects of glycyrrhetinic acid (GA) extracted from Licorice (*Glycyrrhiza glabra*) on the promotion of ROS and reactive nitrogen species (RNS) production. This activation was achieved through the activation of NADPH oxidases and inducible nitric oxide synthase (iNOS), while simultaneously decreasing the production of glutathione (GSH) and the activity of glutathione peroxidase (GPX4). Consequently, these actions led to the exacerbation of lipid peroxidation and the initiation of ferroptosis in triple-negative breast cancer (TNBC) cells [52]. Another study was conducted to examine the impact of a small molecule (DMOCPTL) on GPX4 for the treatment of TNBC. This study utilized an on-target binding assay to evaluate the activity of DMOCPTL. The assay investigating cell death mechanisms revealed DMOCPTL predominantly induces ferroptosis and apoptosis to exert its anti-TNBC effect, accomplished through the ubiquitination of GPX4. The probe employed in the DMOCPTL assay furnished evidence DMOCPTL induced GPX4 ubiquitination by directly interacting with the GPX4 protein [16].

#### 2.6.2. HO-1

HO-1, which is regulated by the transcription factor Nrf2, acts as a critical mediator in ferroptosis, catalyzes the degradation of heme, a pro-oxidant molecule, into biliverdin/bilirubin, carbon monoxide, and ferrous iron. The key role of HO-1 in heme metabolism makes it an important enzyme in ferroptosis. Initially, the release of ferrous iron during this process has the potential to participate in the Fenton reaction, resulting in the generation of ROS. These ROS can subsequently be counteracted by glutathione, a prominent cellular antioxidant. Furthermore, the biliverdin synthesized by HO-1 undergoes swift conversion into bilirubin through the action of biliverdin reductase. Both biliverdin and bilirubin possess robust antioxidant properties, thereby aiding in the safeguarding of cells against oxidative stress. Hence, although HO-1 does not participate directly in the synthesis or recycling of glutathione, it assumes a pivotal role in preserving cellular redox equilibrium and safeguarding cells from oxidative stress, functions that are also fundamental to the glutathione metabolic pathway [100].

However, recent research has revealed a potential downside to HO-1, as its dual functionality appeared to be contingent upon the levels of cellular iron and ROS. In instances of excessive cellular iron and ROS, HO-1 transitions from a protective agent to a perpetrator. This shift is supported by evidence from experiments involving HO-1 knockdown mice and the use of HO-1 inhibitors, which demonstrate HO-1 facilitates erastin-induced ferroptosis and is linked to iron bioavailability [101,102].

Similar to the studies on GPX4, for natural products’ regulation on HO-1, the research also mainly conducted to evaluate the stimulations’ affection on mRNA and protein expression level, mainly up-regulated for treatment or beneficial effects.

#### 2.6.3. NQO1

NQO1 has enzymatic functions catalyzing and reverting a wide variety of compounds, including quinones, nitroaromatic compounds, imidazoles, and iron ions [103,104], and plays a role in ferroptosis.

Study from Xu’s group indicated plumbagin, a potent bioactive compound present in roots, stems, and leaves of the genus Plumbago, could target NQO1/GPX4-mediated ferroptosis to suppress in vitro and in vivo glioma growth [105]. Also, a recent study from Chen’s group demonstrated 2-methoxy-6-acetyl-7-methyljuglone (MAM), a natural naphthoquinone, induced ferroptosis triggered by NQO1 in both chemotherapeutic drug (cisplatin) resistant and targeted drug (AZD9291) resistant NSCLC cells [106].These suggested NQO1 could be a potential target for developing new therapies for gliomas, drug resistant cancers, or other conditions where ferroptosis played a role.

In addition, some research has also shown NQO1 could suppress ferroptosis when it functioned as a coenzyme for tanshinone, a compound from *Salvia miltiorrhiza* (Danshen). Tanshinone helps NQO1 detoxify lipid peroxyl radicals and inhibit ferroptosis both in vitro and in vivo [107]. And, some other quinones derived from *Salvia miltiorrhiza* or *Tabebuia impetiginosa*, which may benefit Alzheimer’s disease (AD), were identified as substrates of these proteins through the NADH decay assay. Notably, the presence of NQO1 or FSP1 resulted in an observed augmentation of anti-lipid peroxidation activity in the liposomes [99].

#### 2.6.4. NOX4

The generation of superoxide anion and reactive oxygen species by NADPH oxidase (NOX), through the consumption of NADPH, represents a significant contributor to oxidative stress. The overexpression of NOX results in the depletion of NADPH, the accumulation of intracellular oxidative stress, and heightened vulnerability to ferroptosis. Conversely, NOX expression or activity inhibition can confer resistance or insensitivity to ferroptosis upon cells. NOX4 is one of the members of the NADPH oxidase family. Research has shown NOX4 is associated with both immunity and ferroptosis [108]. In the context of specific diseases, NOX4 has been found to promote ferroptosis of astrocytes by oxidative stress-induced lipid peroxidation, and a previous study reported a high expression of NOX4 was associated with poor prognosis in colon cancer, indicating NOX4 may act as a biomarker for colon cancer ferroptosis [109]. But the studies on the effect of natural products on NOX4 are limited.

Tectorigenin, a main component derived from *Belamcanda chinensis*, has proved to possess multiple pharmacological activities, including improving kidney injury caused by ferroptosis by mediating the oxidative stress response. In the in vitro investigation on chronic kidney disease treatment, tectorigenin stimulation significantly inhibited the transcription and protein level of NOX4, indicating tectorigenin could confer resistance or insensitivity to ferroptosis [81].

#### 2.6.5. GCLC and GCLM

GCLC and GCLM are two subunits of glutamate-cysteine ligase (GCL), the enzyme responsible for limiting the rate of glutathione (GSH) synthesis, a crucial antioxidant in cellular function [102]. Prior research has demonstrated the knockout of GCLC in mice resulted in a complete deficiency of GSH and embryonic mortality, while the knockout of GCLM led to a significant reduction in cellular GSH levels.

The reported natural products’ regulation on GCLC and GCLM mainly focused on cancer treatment (including colorectal cancer, esophageal cancer, and gastric cancer), or diabetic retinopathy (DR). For treating colorectal cancer, andrographolide, or andrographis extract, derived from *Andrographis paniculata*, all showed potential in treating colorectal cancer, potential mechanisms including upregulation of GCLC and GCLM at mRNA level, both in vitro and in vivo [95,96]. Andrographolide also showed potential in gastric cancer in vitro by altering HMOX1, GCLC, and GCLM gene expression [93]. In the context of esophageal cancer treatment, it has been observed Oridonin (Ori) possessed the capability to establish a covalent bond with cysteine, resulting in the formation of a conjugate referred to as oridonin-cysteine (Ori-Cys). This interaction leads to the inhibition of glutathione synthesis, which aligns with the observed decline in the enzymatic activity of GCLC. Consequently, there is a reduction in the intracellular ratio of GSH/GSSG, accompanied by a significant decrease in the enzymatic activity of GPX4 [80]. For diabetic retinopathy (DR), astragaloside-IV (AS-IV) could increase the expression of GPX4, GCLC, and GCLM in vitro [86].

### 2.7. The Effects of Natural Products on Enzymes Involving Lipid Metabolism

ACSL3 and ACSL4 are enzymes that belong to the long-chain fatty acyl CoA synthetase family (ACSLs), which contribute significantly to lipid metabolism [30]. ACSL4 is a positive regulator in ferroptosis. On the other hand, ACSL3 contributes to cancer cells acquiring resistance to ferroptosis. This means that while ACSL4 promotes ferroptosis, ACSL3 helps cancer cells resist this process [110].

*Epimedium koreanum* Nakai, one of the species of Epimedii Folium, is widely used worldwide as an herbal supplement but has a severe propensity for hepatotoxicity. The in vivo study investigating the mechanism of its hepatotoxicity identified the ferroptosis-promoting protein ACSL4 was significantly up-regulated, and GPX4 and System Xc^−^ were significantly down-regulated, indicating the mechanism of hepatotoxicity of *Epimedium koreanum* Nakai may related to the induction of ferroptosis in hepatocytes [82].

Icariin (ICA), a compound derived from Epimedii, may be responsible for the plant’s toxicity, the results obtained from both in vitro and in vivo experiments demonstrated ICA exerted a significant down-regulatory effect on the protein expression of PPAR-α. Additionally, it down-regulated the expression of APOB, ACSL3, ACSL4, and up-regulated 5/12/15-HETE, indicating the involvement of a lipid metabolism disorder in the nephrotoxicity induced by ICA. Furthermore, ICA was observed to enhance the accumulation of iron and levels of lipid peroxidation (LPO), while simultaneously reducing the activity of GPX4 [83]. In the contrast, ICA also shared the beneficial effect on myocardial hypoxia/reoxygenation (H/R) injury, since the in vitro study demonstrated a significant reduction in mRNA expressions and protein levels of Nrf2 and HO-1 following H/R stimulation, which were subsequently elevated with increasing concentrations of ICA. Additionally, the expression of ACSL4 decreased with increasing ICA concentration, while the expression of GPX4 increased [76].

Except for above research focused on the regulation of the mRNA or protein expression on ACSL3 or ACSL4, there is also a study investigating the enzymatic activity of ACSL4. Silibinin (SIL), a compound derived from *Silybum marianum*, exhibited a protective effect on HepG2 cells against ferroptosis, which was found to be dependent on ACSL4. To validate this interaction, biophysical assays and a SIL derivatized chemical probe were employed, confirming the binding capability of SIL to ACSL4. Subsequent enzymatic assays demonstrated SIL effectively inhibited the enzymatic activity of ACSL4, consequently attenuating the ACSL4-mediated ferroptosis [98].

## 3. Materials and Methods

### 3.1. Data Retrieval

The Web of Science^TM^ (Clarivate^TM^, London, UK) platform was used for literature survey to achieve a dataset of studies reporting the effects of natural products on enzymes contributing to ferroptosis. The search query was performed in WOS core collection based on keywords: ferroptosis, enzyme, and herbal product. Then, we conducted the search strategy: All Fields (ferroptosis) AND All Fields (herb* OR herbal-products OR herbal-supplements OR natural-supplements OR botanical-supplements OR phytotherapy OR dietary-supplementation OR plant-extract OR traditional-medicine OR natural-product OR Botanical*), and in life/medical science focused.

### 3.2. Screening and Eligibility

According to the selection criteria outlined in the preferred reporting items for systematic reviews and meta-analyses (PRISMA) guidelines [111,112], publications were retrieved from the WOS database, and all search results were compiled and systematically reviewed, with each source being carefully evaluated and labeled as either included or excluded in an excel spreadsheet. Initially, duplicates were identified and removed. Subsequently, eligibility criteria were established prior to the commencement of this review. As shown in Table 3.

### 3.3. Annotated Bibliography

Upon completion of the initial screening process, all papers meeting the eligibility criteria were downloaded. Subsequently, we meticulously examined the abstract or entire text of each research article to ascertain its continued relevance. Furthermore, we annotated each eligible research article by manually incorporating the retrieved information, encompassing the natural products investigated in each paper (i.e., ingredients, including what Chinese medicine or natural product the compound is derived from), herbal extracts, or formulae/preparations.), the tested enzymes, the cell line, the animal model used in each study, the biomolecules involved in each study, the specific mechanism of action discovered in each study, and the diseases that concerned each study. The statistical data were then generated to describe the current status of research in this field, such as the disease, natural products that were commonly concerned, and the enzymes that were most likely affected.

## 4. Conclusions

Ferroptosis is characterized as a controlled mechanism of membrane degradation resulting from the impairment of the lipid redox protection system and the disruption of iron homeostasis. Notably, ferroptosis possesses a dual nature, capable of either eliminating pathological cells to uphold bodily equilibrium and continuity, or inflicting harm upon normal cells, leading to illness. Exogenous stimuli and substances, including various natural products, exert distinct effects on the promotion or prevention of ferroptosis depending on the specific pathological context.

Up to now, the majority of research has concentrated on investigating the impact of natural products on the expression of enzymes involving ferroptosis, while limited attention has been given to the regulation of enzyme activity. This observation underscores the considerable potential and scope for exploring the influence of natural products on enzyme activity. Through extensive screening and analysis of various natural products, this review has provided several promising substances that exhibit noteworthy regulatory effects, elucidating their underlying mechanisms and modes of action. These findings offer novel insights and a foundation for future advancements and applications in the field of natural product development and utilization.

## Figures and Tables

**Table 1:** The enzymes involved in ferroptosis.

Pathway	Effect on Ferroptosis	Gene Name	Uniprot ID	Protein Name	Ref.
Redox homeostasis	Inhibit	GPX4	P36969	Glutathione peroxidase 4	[15,16]
		NQO1	P15559	NAD(P)H Quinone Dehydrogenase 1	[17]
	Promote	NOX1	Q9Y5S8	NADPH oxidase 1	[18]
		NOX4	Q9NPH5	NADPH oxidase 4	[19]
		ALOX5	P09917	Polyunsaturated fatty acid 5-lipoxygenase	[20]
		DPP4	Q9NPH5	Dipeptidyl peptidase 4	[21]
Iron homeostasis/redox homeostasis	Heme metabolism	HMOX1	P09601	Heme oxygenase 1	[22,23]
GSH homeostasis	Inhibit	GCLC	P48506	Glutamate-cysteine ligase catalytic subunit	[24]
		GCLM	P48507	Glutamate-cysteine ligase regulatory subunit	[24]
	Promote	CARS	P49589	Cysteine-tRNA ligase, cytoplasmic	[25]
		CHAC1	Q9BUX1	Glutathione-specific gamma-glutamylcyclotransferase 1	[26]
Lipid metabolism	Inhibit	HELLS	Q9NRZ9	Lymphoid-specific helicase	[27]
		SCD	O00767	Stearoyl-CoA desaturase	[20]
		FADS2	O95864	Acyl-CoA 6-desaturase	[28]
	Promote	ACSL4	O60488	Acyl-CoA Synthetase long chain family member 4	[29]
		ACSL3	O95573	Acyl-CoA Synthetase long chain family member 3	[30]
		LPCAT3	Q6P1A2	Lysophosphatidylcholine acyltransferase 3	[31]
		CS	O75390	Citrate synthase	[32]
Glucose metabolism	Inhibit	PHGDH	O43175	D-3-phosphoglycerate dehydrogenase	[33]
		G6PD	P11413	Glucose-6-phosphate 1-dehydrogenase	[34]
	Promote	PHKG2	P15735	Phosphorylase kinase subunit gamma-2	[35]
Mevalonate pathway	Promote	SQLE	Q14534	Squalene monooxygenase	[36]
		FDFT1	P37268	Squalene synthase	[37]

**Table 2:** Natural products’/phytochemicals’ regulation on enzymes involving ferroptosis.

Enzyme	Natural Product (Compound)	Natural Product (Herb /Chinese Medicine)	Cell Line	Animal	Activity/Mechanism	Relevant Disease	Ref.
GPX4	Baicalein (5,6,7-trihydroxyflavone)	*Solanum nigrum* L.	PANC1, BxPc-3	— —	At the protein level, baicalein suppresses erastin-mediated degradation of GPX4.	Ferroptosis-associated tissue injury	[42]
GPX4	Jiyuan oridonin A derivative (a2)	Jiyuan *Rabdosia rubescens*	HGC-27, MGC-803, BGC-823, AGS, GES1	Gastric cancer cell line-derived xenograftmicemodels	Compound a2 decreased GPX4 expression	Gastric cancer	[43]
GPX4	Artemisia santolinifolia ethanol extract (ASE)	*Artemisia santolinifolia*	A549 and H23	— —	ASE decreased the GPX4 level at a significantly higher rate in ASE concentrations of 200 µg/mL.	Non-small cell lung cancer (NSCLC)	[44]
GPX4	Kayadiol	*Torreya nucifera*	NKTCL	— —	Kayadiol decreased the expression of GPX4.	Cancer	[45]
GPX4	DMOCPTL	A derivative of natural product parthenolide	MDAMB-231, SUM159, BT574,4T1, Hs578T, MDA-MB-468	Mouse	DMOCPTL directly binding to GPX4 protein, inducing GPX4 ubiquitination.	Triple-negative breast cancer (TNBC)	[16]
GPX4	— —	Guizhi Fuling Capsule (GFC)	— —	C57BL/6 mice	GFC could inhibit the expression of GPX4 at protein level.	Gynecological diseases	[46]
GPX4	Isothiocyanate sulforaphane	Cruciferous vegetables	U-937, MV4-11	— —	Isothiocyanate sulforaphane decreased GPX4 protein expression level.	Acute myeloid leukemia	[47]
GPX4	Resveratrol (RSV)	— —	MIN6	— —	RSV could increase the expression of GPX4, and decrease the expression of ACSL4.	Insulin secretion dysfunction	[48]
GPX4	Arsenic	Realgar	HK2	Mouse	Realgar reduced expression of SLC7A11 and GPX4.	Nephrotoxicity	[49]
GPX4	Aristolactam I (ALI)	*Aristolochia* and *Asarum* plants	mRTECs	Wild-type C57BL/6 mice and kidney-specific Rev-erbα knockout mice with AAI-induced nephropathy	ALI treatment led to decreased GPX4 in mRNA and protein level.	Nephropathy	[50]
GPX4	Heteronemin	*Hippospongia* sp.	HA22T/VGH, HA59T	— —	Heteronemin treatment reduced the expression of GPX4.	Hepatocellular carcinoma (HCC)	[51]
GPX4	Glycyrrhetinic acid (GA)	Licorice (*Glycyrrhiza glabra*)	MDA-MB-231, BT-549, and MCF-10A	— —	GA down-regulated the expression of SLC7A11 at protein level; GA treatment (40 μM) significantly inhibited the activity of GPX4 but no influence on GPX4 expression.	Triple-negative breast cancer (TNBC)	[52]
GPX4	Pratensein (PTS)	*Trifolium pretense* L.	H9c2	— —	PTS treatment up-regulated Nrf2 expression and GPX4 expression at protein level in OGD/R-treated H9c2 cells.	Myocardial infarction (MI), myocardial ischemia-reperfusion (I/R) injury	[53]
GPX4	Cryptotanshinone (CTN)	*Salvia miltiorrhiza* Bunge (Danshen)	A549, NCI-H520, and BAES-2B	— —	CTN can inhibit the activity of GPX4.	Lung cancer	[54]
GPX4	Pentacyclic triterpenoids (PTs), including glycyrrhetinic acid (GA), ursolic acid (UA) and oleanolic acid (OA)	PTs are natural products that can be found in various plants, such as licorice, rosemary, olive, and loquat.	HCT116, HeLa, A549, L02, AT-II, and HEK293	Nude mice with HCT116 tumor xenografts	GA treatment led the ferroptosis negative regulatory protein expression (GPX4, SLC40A1, and SLC7A11) decreased considerably.	Colorectal cancer (CRC)	[55]
GPX4	Astragaloside IV (Ast-IV)	*Astragalus membranaceus* Bunge, a traditional Chinese herb	— —	C57BL/6J male mice, lung injury model induced by PM2.5	The Ast-IV intervention increased the expression of GPX4, which was decreased in PM2.5 group.	Lung injury caused by PM2.5 exposure	[56]
GPX4	Wogonoside, wogonin, palmatine, paeoniforin and liquiritin	Shaoyao Decoction (SYD)	Caco-2	Sprague–Dawley male rats with TNBS-induced colitis model	SYD induced activation of GPX4 transcription and increase of protein expression.	Inflammatory bowel disease (IBD), especially ulcerative colitis (UC)	[57]
GPX4	Polyphenols sourced from Ilex latifolia Thunb. (PIT)	*Ilex latifolia* Thunb.	Porcine intestinal epithelial cells (IPEC-J2)	Weanling piglets under oxidative stress induced by diquat injection	Supplementation with PIT significantly reduced jejunal TFR1 gene expressions and improved SLC7A11 and GPX4 gene expressions in the piglets under oxidative stress.	Intestinal injury caused by oxidative stress	[58]
GPX4	Danshensu (Dan)	Dan is a pure molecule derived from the root of the *Salvia miltiorrhiza* herb, Danshen.	LX-2 human and T6 rat hepatic stellate cells (HSCs)	— —	Dan up-regulated GPX4, and SLC7A11.	Liver fibrosis and cirrhosis	[59]
GPX4	— —	Naotaifang extract (NTE): a compound traditional Chinese herbal medicine composed of four herbs: Radix Astragali (Huangqi), Rhizoma chuanxiong (Chuangxiong), Pheretima (Dilong), and *Bombyx batryticatus* (Jiangcan)	— —	Sprague-Dawley (SD) rats with middle cerebral artery occlusion (MCAO) model of acute cerebral ischemia	NTE increased the expression levels of SLC7A11, GPX4.	Ischemic stroke	[60]
GPX4	Cucurbitacin B (CuB)	*Trichosanthes kirilowii* Maximowicz	Human nasopharyngeal carcinoma CNE1 cells	BALB/c nude mice, human nasopharyngeal carcinoma murine xenograft model	CuB induced ferroptosis by increasing lipid peroxidation and reducing the expression of GPX4 in mRNA and protein level.	Nasopharyngeal carcinoma	[61]
GPX4	Dihydroartemisinin (DHA) and Chlorin e6 (Ce6)	DHA derived from the natural plant *Artemisia annua*, is a sesquiterpene lactone compound	Lewis cells (LLC), a lung cancer cell model	— —	DHA could reduce GPX4 at mRNA and protein level.	Lung cancer	[62]
GPX4	Perillaldehyde	*Ammodaucus leucotrichus* Coss. and Dur (*A. leucotrichus*), commonly known as hairy cumin	HL-60, Jurkat, DLD-1, SHSY5Y	— —	Perillaldehyde decreased GPX4 protein expression.	Acute myeloid leukemia	[63]
GPX4	*Lycium barbarum* polysaccharide (LBP)	The fruits of the traditional Chinese herb *L. barbarum*.	MCF-7, MDA-MB-231	— —	LBP down-regulated the protein level of xCT (SLC7A11) and GPX4.	Breast cancer	[64]
GPX4	Matrine	*Sophora flavescens*	HCT116	— —	Matrine down-regulated the protein level of GPX4.	Colorectal cancer (CRC)	[65]
GPX4	Wogonoside (WG)	*Scutellaria baicalensis* Georgi, a perennial herb of the Labiatae family	HSC-T6, AML-12, RAW264.7	C57BL/6 mice, induced by CCl4 for liver fibrosis model	WG down-regulated the protein levels of GPX4 and SLC7A11.	Liver fibrosis	[66]
GPX4	Suberitenones A and B, mycalols	Hemimycale topsenti, Haliclona (Rhizoniera) dancoi	HepG2, A549, A2058, MRC5	— —	Mycalols could reduce the level of GPX4 protein in HepG2 cells.	Hepatocellular carcinoma, lung carcinoma, melanoma, anaplastic thyroid carcinoma	[67]
GPX4	Polyphyllin VI (PPVI)	*Paris polyphylla*	HCCLM3, Huh7 and THLE-2	Male BALB/c nude mice with subcutaneous tumor model of Huh7 cells	The GPX4 protein expression levels in the HCCLM3 and Huh7 cells were down-regulated, and negatively associated with the concentration of PPVI.	Hepatocellular carcinoma (HCC)	[68]
GPX4	6-Gingerol	Ginger (Zingiber officinale Roscoe)	A549	BALB/cNude mice, A549 tumor xenografts	6-Gingerol down-regulate the expressions of GPX4 at protein level.	Lung cancer	[69]
GPX4	Realgar (REA)	— —	Eca109, KYSE150	— —	REA IC50 and 2IC50 caused significant down-regulation of GPX4.	Esophageal cancer	[70]
GPX4, HO-1	Eriodictyol	Citrus fruits and some Chinese herbal medicines	HT-22	APPswe/PS1E9 transgenic mice, a mouse model of Alzheimer’s disease	Eriodictyol treatment could increase the GPX4, and HO-1 at protein level in both the cortex and hippocampus of APP/PS1 mice.	Alzheimer’s disease (AD)	[71]
GPX4, HO-1	β-elemene	*Curcumae rhizoma*	HCT116, LoVo, CaCO2		β-elemene in combination with cetuximab was shown to upregulate the HO-1 and down-regulate GPX4.	Colorectal cancer	[72]
GPX4, HO-1	— —	*Tripterygium wilfordii* Hook.f. (TwHF)	— —	APPswe/PS1E9 transgenic mice, a mouse model of Alzheimer’s disease	TwHF treatment reduced the PQ up-regulated HO-1 level, and it up-regulated the expression of GPX4 compared to the PQ group.	Alzheimer’s disease (AD)	[73]
GPX4, HO-1	FPHLP (low-polarity fraction from Ficus pandurata Hance)	*Ficus pandurata* Hance	HepG2	C57BL6/J mice, CCl4-induced acute liver injury model	FPHLP significantly reduced the level of Fe^2+^ and expression of TfR1, xCT/SLC7A11, and Bcl2, while increasing the expression of GPX4.	Acute liver injury	[74]
GPX4, HO-1	Biochanin A (BCA)	Huangqi	— —	C57BL/6 mice	The tendency of HO-1, and GPX4 expression was downregulated when treated with ferric ammonium citrate and upregulated after incubation with BCA.	knee osteoarthritis	[75]
GPX4, HO-1, ACSL4	Icariin (ICA)	*Epimedium*	H9C2	— —	The mRNA expressions and protein levels of Nrf2 and HO-1 were reduced notably after H/R stimulation but were elevated with the increase of ICA concentration. The expression of ACSL4 were decreased with the increase of ICA concentration, while the expression of GPX4 was increased with the increase of ICA concentration.	Myocardial ischemia /reperfusion injury	[76]
GPX4, HO-1	Nobiletin	Citrus peel	SK-MEL-28	— —	The mRNA and protein level of GPX4 and HO-1 was decreased in nobiletin-treated melanoma cells.	Melanoma	[77]
GPX4, HO-1	— —	GBH (Insamgobonhwan)	HT22	ICR mouse	GBH can reverse the RSL3 induced GPX4 reduction and HO-1 increase.	Alzheimer’s disease (AD)	[78]
GPX4, HO-1	Salvianolic acid B(Sal B)	*Salvia miltiorrhiza* (Danshen in Chinese)	H9C2	Sprague-Dawley rats, myocardial infarction model	Nrf2 was strongly activated in MI rats pretreated with Sal B, and resulted in the upregulation of its target genes including HO-1, xCT, Gpx4, Fth1, and Fpn1.	Myocardial infarction (MI)	[79]
GPX4, GCLC	Oridonin (Ori)	*Rabdosia rubescens* (Hemsl.)	TE1	— —	Ori can decrease the enzymatic activity of GCLC and GPX4.	Esophageal cancer	[80]
GPX4, NOX4	Tectorigenin	*Belamcanda chinensis*	Primary renal tubular epithelial cells	— —	Tectorigenin treatment greatly inhibited Smad3 phosphorylation, and the transcription and protein level of Nox4; while it indirectly restored the expression of GPX4.	Chronic kidney disease	[81]
GPX4, ACSL4	— —	*Epimedium koreanum* Nakai	— —	Rat	*Epimedium koreanum* Nakai ethanol extract (EEE) downregulated GPX4, and up-regulated ACSL4 significantly.	Toxicity-natural products-induced liver injury	[82]
GPX4, ACSL3, ACSL4	Icaritin	*Epimedium*, a traditional Chinese medicine	HK-2 cells, hOAT1-HEK293 cells, and mock-HEK293 cells	C57BL/6 mice, with icaritin-induced nephrotoxicity model	Icaritin significantly downregulated the protein expression of ACSL3, and ACSL4, and it can reduce the activity of GPX4.	Icaritin-induced nephrotoxicity	[83]
GPX4, ACSL4	Gossypol acetic acid (GAA)	The seeds of cotton plants	H9C2 cardiomyoblast cells and neonatal rat cardiomyocytes	Sprague-Dawley	GAA significantly decreased the mRNA levels of Ptgs2 and Acsl4, decreased the protein levels of ACSL4 and NRF2, and increased the protein levels of GPX4 in I/R-induced ex vivo rat hearts.	Cardiac Ischmia/Reperfusion Injury	[84]
GPX4, ACSL4	— —	Xiaoyaosan: Radix Angelicae sinensis, Radix *Paeoniae alba*, Poria, Radix bupleuri, Rhizoma *Atractylodis macrocephalae*, Radix glycyrrhizae, *Herba menthae*, Rhizoma Zingiberis recens	— —	C57BL/6	The Xiaoyaosan treatment could upregulate the stress-induced down-regulation of GPX4; and down-regulate the stress-induced up-regulation of ACSL4.	Depressive-like behavior	[85]
GPX4, GCLC, GCLM	Astragaloside-IV (AS-IV)	*Astragalus membranaceus*	ARPE-19 cells	— —	AS-IV could restore the down-regulated expression of GPX4, GCLM, and GCLC under high glucose conditions.	Diabetic retinopathy (DR)	[86]
HO-1	Panaxydol (PX)	Panax ginseng	BEAS-2B	Specific pathogen-free (SPF) male C57BL/6 mice, murine model of LPS-induced ALI.	PX increased Nrf2 and HO-1 expression in comparison with LPS group.	Acute lung injury (ALI)/acute respiratory distress syndrome (ARDS)	[87]
HO-1	Tagitinin C	*Tithonia diversifolia*	HCT116	— —	Tagitinin C increased mRNA expression level of HO-1	Colorectal cancer	[22]
HO-1	Nodosin	*Isodon serra* (Maxim.) Kudo	T24, UMUC3, SW780	Xenograft tumor model	Nodosin up-regulated the expression of genes, including HMOX1, G0S2, SQSTM1, FTL and AIFM2	Muscle-invasive bladder cancer (MIBC)	[50,88]
HO-1	— —	Propolis	SU-DHL-2	— —	The expressions of HO-1 were up-regulated at mRNA level when treated by propolis.	Diffuse large B-cell lymphoma (DLBCL)	[89]
HO-1	Thonningianin A (Th A)	*Penthorum chinense* Pursh.	SH-SY5Y	Zebrafish (AB strain of wild-type zebrafish); 6-OHDA-induced zebrafish model of Parkinson’s disease	Th A treatment significantly facilitated the Nrf2 nuclear translocation and subsequently increased the HO-1 protein level.	Parkinson’s disease (PD)	[90]
HO-1	Icariin (ICA)	*Herba epimedii*	Endplate chondrocytes, nucleus pulposus cells, annulus fibrosus cells	C57BL/6J	ICA treatment led to notably increased protein levels of Nrf-2 and its downstream HO-1.	Intervertebral disc degeneration	[91]
HO-1	*Astragalus membranaceus*, Astragaloside IV, Astragalus polysaccharide, Swainsonine, Daidzein	*Astragalus membranaceus*, a kind of traditional Chinese medicine	HepG2	— —	miRNA level of HMOX1 can be up-regulated by *Astragalus membranaceus*.	Hepatocellular carcinoma (HCC)	[92]
HO-1, GCLC, GCLM	Andrographolide	*Andrographis paniculata*, a traditional Chinese herbal medicine	MKN74 and NUGC4	— —	Andrographis treatment upregulated the expression of HMOX1, GCLC, and GCLM	Gastric cancer	[93]
HO-1, NQO1	Full-fat rice bran	A by-product of rice processing.	— —	Yorkshire pigs were used as experimental animals to establish a prolonged cold stimulation model.	Full-fat rice bran promoted the mRNA expression of Nrf2 and NQO1, as well as the protein content of Nrf2 and HO-1.	Cardiac injury and energy metabolism disturbance caused by prolonged cold stimulation.	[94]
HO-1, GCLC, GCLM	Andrographolide	*Andrographis paniculata*, a traditional herb	HCT116, HT29, NCM460	athymic nude mice, subcutaneous xenograft model	HMOX1, GCLC, and GCLM were significantly up-regulated in the andrographis and the combination treatment group vs. the untreated group.	Colorectal cancer (CRC)	[95]
HO-1, GCLC, GCLM	Andrographis extract (standardized to 20% andrographolide content)	*Andrographis paniculata*	HCT116 and SW480	Mouse	Andrographis could upregulate the HMOX1, GCLC, and GCLM, in mRNA and protein level individually or in combination with 5FU.	Colorectal cancer (CRC)	[96]
ACSL4	β-Elemonic acid (EA)	EA is isolated from Boswellia papyrifera, a plant used in traditional medicine.	CRC lines SW480, HCT116 and HT29; the control colorectal cell line NCM460	Female BALB/c nude mice; subcutaneous xenograft model of CRC	EA at high concentration induces ferroptosis by downregulating FTL and upregulating TF, CP, and ACSL4.	Colorectal cancer (CRC)	[97]
ACSL4	Silibinin (SIL)	*Silybum marianum*	HepG2, HEK293T, Hep1-6	— —	The enzymatic assays showed SIL inhibited ACSL4 enzymatic activity, thereby mitigating the ACSL4-mediated ferroptosis.	Liver diseases	[98]
NQO1	Quinones: Cryptotanshinone, idebenone, menaquinone 4, dopamine, lipoic acid, ascorbic acid, baicalein, 3,3′-diindolylmethane, fisetin, triptophenolide, cynaroside (luteoloside), icaritin, gossypol, carnosol, honokiol, β-lapachone, dihydroisotanshinone I (DHIT I), dihydrotanshinone I (DHT I), tanshinone I and cryptotanshinone.	— —	HT22, MC65	— —	All quinones that were substrates of these proteins as identified by the NADH decay assay displayed an increase in anti-lipid peroxidation activity in the liposomes in the presence of NQO1 or FSP1.	Alzheimer’s disease (AD)	[99]

**Table 3:** Eligibility criteria of selected articles.

No.	Eligibility Criteria
1	Not included article types. e.g., review, proceedings, feature, editorial material.
2	Not in the life Sciences
3	Irrelevant object/topic
4	Full text not available

## Data Availability

The data presented in this study are contained within the article.
